# Antioxidant and Anticholinesterase Potential of Six *Thymus* Species

**DOI:** 10.1155/2015/403950

**Published:** 2015-08-16

**Authors:** Marija Kindl, Biljana Blažeković, Franz Bucar, Sanda Vladimir-Knežević

**Affiliations:** ^1^Department of Pharmacognosy, Faculty of Pharmacy and Biochemistry, University of Zagreb, Marulićev trg 20, 10000 Zagreb, Croatia; ^2^Department of Pharmacognosy, Institute of Pharmaceutical Sciences, University of Graz, Universitätsplatz 4/I, 8010 Graz, Austria

## Abstract

The present study aimed to evaluate antioxidant and acetylcholinesterase (AChE) inhibitory activities of the ethanolic extracts of six selected *Thymus* species growing in Croatia (*T. longicaulis*, *T. praecox* subsp. *polytrichus*, *T. pulegioides*, *T. serpyllum* subsp. *serpyllum*, *T. striatus*, and *T. vulgaris*). Antioxidant effectiveness was assessed using six different assays, in comparison with rosmarinic acid, luteolin, and reference antioxidants. All tested *Thymus* extracts possessed DPPH (IC_50_ = 3–6 *μ*g/mL) and nitric oxide (IC_50_ = 70–177 *μ*g/mL) free radical scavenging activities, strong reducing properties (IC_50_ = 11–15 *μ*g/mL), ferrous ion chelating activity (IC_50_ = 126–389 *μ*g/mL), ability to inhibit lipid peroxidation (IC_50_ = 34–80 *μ*g/mL), and high total antioxidant capacities (238–294 mg AAE/g). AChE inhibitory activity was examined using Ellman's colorimetric method and all tested extracts showed anti-AChE activity in a dose dependent manner. The values of 10–28%, 23–39%, and 64–86% were obtained for tested concentrations of 0.25, 0.5, and 1 mg/mL, respectively. Additionally, the contents of total hydroxycinnamic derivatives, flavonoids, and tannins in dried plant samples were determined spectrophotometrically. Our results highlighted *Thymus* species as a rich source of natural antioxidants and AChE inhibitors that could be useful in preventing and treating Alzheimer's disease and other neurodegenerative disorders.

## 1. Introduction

In recent years, natural antioxidants from medicinal plants have been intensively investigated in order to find compounds capable of protecting against a number of diseases related to oxidative stress and free radical-induced damage. Oxidative stress, as an imbalance between production and removal of free radicals and other reactive species, is well known to cause the oxidation of important biomolecules leading to cellular damage and death. Therefore, oxidative stress plays critical role in the pathogenesis of various diseases, including cardiovascular, neurodegenerative, and inflammatory diseases, cancer, and diabetes, as well as aging processes. Aiming to neutralize the deleterious effects of free radicals and reactive oxygen and nitrogen species, different antioxidant strategies have involved enhancement of enzymatic or nonenzymatic defense mechanisms through dietary or pharmacological means [1]. Plant polyphenols, as being widely distributed secondary metabolites in plant kingdom and abundant in our diet, are considered to be one of the most important classes of phytochemicals. Nowadays, polyphenols have gained a lot of importance because of their potential use as prophylactic and therapeutic agents in many diseases, and much work has been presented by the scientific community which focuses on their antioxidant effects [2]. Polyphenols can prevent that oxidative damage acting as free radical scavengers, reducing agents, chelating transition metals to suppress the initiation of radical formation, inhibiting lipid peroxidation, and regulating defense enzymes, as well as modulating cell signaling pathways and gene expression [3].

Alzheimer's disease (AD) as the most common cause of dementia is a progressive age-related neurodegenerative disorder that is characterized by loss of memory and cognitive abilities which significantly interfere with person's daily life. Characteristic neuropathological findings include selective neuronal and synaptic losses, extracellular neuritic plaques containing the amyloid *β*-peptide, and intracellular neurofibrillary tangles composed of hyperphosphorylated forms of the tau protein, as well as accumulation of transition metals such as copper, zinc, and iron [4–6]. Despite decades of research and advances in understanding of AD pathogenesis, current pharmacotherapeutic options are still very limited and represent an area of significant unmet need. One of the most used therapeutic strategies in the AD treatment, based on the cholinergic hypothesis, is the use of inhibitors of acetylcholinesterase (AChE), the principal enzyme involved in the hydrolysis of acetylcholine in central cholinergic synapses. Inhibition of AChE also serves as a strategy for the treatment of senile dementia, ataxia, myasthenia gravis, and Parkinson's disease [7]. The present drugs with AChE inhibitory activity are effective only against mild to moderate type of AD, provide only temporary symptomatic relief, and possess some considerable side effects related to cholinergic stimulation in brain and peripheral tissues [8]. Hence, the search for new sources of antiacetylcholinesterase agents which are effective, selective, and with less negative effects has increased greatly in recent years. Previous studies have shown that various plant extracts and their bioactive compounds provide promising alternatives to current therapies for neurodegenerative disorders [9]. Increased oxidative stress and impaired cellular energy metabolism are characteristics of many important age-related diseases, and AD is no exception. Cells in the brains of AD patients exhibit abnormally high amounts of oxidatively modified proteins, lipids, and DNA. Such free radical-mediated molecular damage is particularly prominent in the environment of plaques and in neurofibrillary tangle-bearing neurons suggesting roles for oxidative stress in AD [6]. In this regard, it is important for any drug candidate, which can be used for AD treatment, to possess cholinesterase inhibitory potential and antioxidant activity.

Among the aromatic plants belonging to the Lamiaceae family, the genus* Thymus* is noteworthy for the numerous species and varieties of wild growing plants.* Thymus* species are perennial, aromatic herbs, and subshrubs native to Europe, North Africa, and Asia. They are commonly used as culinary herbs and flavouring agents. Because of their antimicrobial, spasmolytic, and antioxidant effects, they are also used for medicinal purposes [10]. Also, anti-inflammatory, antinociceptive, and antitumor activities of* Thymus* species have been reported [11, 12]. The important representatives of genus* Thymus* wild growing in Croatia, studied in this paper, are* T. longicaulis* C. Presl,* T. praecox* Opiz subsp.* polytrichus* (A. Kerner Ex Borbás) Jalas,* T. pulegioides* L.,* T. serpyllum* L. subsp.* Serpyllum*, and* T. striatus* Vahl. They are known as a traditional medicine for the treatment of cold, flu, cough, headache, neurosis, stomach diseases, nephritis, and high cholesterol [13–15]. Most of the previous studies of these plants were focused on volatile compounds while other bioactive components and their biological effects have not yet been fully investigated. Due to the lack of scientific evidence, the present study aimed to evaluate antioxidant and anti-AChE potential of the ethanolic extracts of selected wild growing Croatian* Thymus* species in comparison with* T. vulgaris*. Additionally, the contents of different polyphenolic components, hydroxycinnamic acids, flavonoids, and tannins were determined in all studied plants.

## 2. Materials and Methods

### 2.1. Plant Material

Aerial parts of five investigated* Thymus* species were collected at the full flowering stage in June and July 2011 from different locations in Croatia:* Thymus longicaulis* C. Presl (Jasenice, 130 m a.s.l.),* Thymus praecox* Opiz subsp.* polytrichus* (A. Kerner Ex Borbás) Jalas (Veliki Alan, 1340 m a.s.l.),* Thymus pulegioides* L. (Ivanščica, 900 m a.s.l.),* Thymus serpyllum* L. subsp.* serpyllum* (Đurđevački pjesci, 110 m a.s.l.), and* Thymus striatus* Vahl (Buljma, 1450 m a.s.l.). The commercial sample of Thymi herba (*Thymus vulgaris* L.) was obtained from Jan Spider (Pitomača, Croatia). All plant samples were identified at the Department of Pharmacognosy, Faculty of Pharmacy and Biochemistry and Department of Botany and Botanical Garden, Faculty of Science (University of Zagreb, Croatia) where the voucher specimens have been deposited under the genus number 819.

### 2.2. Preparation of the Extracts

Air-dried and pulverized plant material (10.00 g) was extracted with 70% ethanol (100 mL) using an ultrasonic bath for 30 min. The extract was filtered using a Büchner funnel and the residue was then reextracted with the same solvent (100 mL) as described above. Obtained extracts were combined and then concentrated to dryness under reduced pressure at 50°C using a rotary evaporator. The extraction yields (w/w) are given in Table 1.

### 2.3. Chemicals

Acetylcholinesterase (AChE) from electric eel (Type VI-S, EC 3.1.17.), acetylthiocholine iodide (ATChI), bovine brain extract (Folch type VII), 3-(2-Pyridyl)-5,6-diphenyl-1,2,4-triazine-4′,4′′-disulfonic acid (ferrozine), 2,2-diphenyl-1-picrylhydrazyl (DPPH^•^), 5,5′-dithiobis(2-nitrobenzoic acid) (DTNB), galantamine hydrobromide, sulfanilamide, and rosmarinic acid (96%) were obtained from Sigma-Aldrich (St. Louis, MO, USA). Aluminium chloride, ethylenediaminetetraacetic acid (EDTA), and hexamethylenetetramine were purchased from Kemika (Zagreb, Croatia). Butylhydroxytoluene (BHT, ≥99%), luteolin (≥97%), N-(1-naphthyl)ethylenediamine, sodium nitroprusside, and trolox (≥98%) were purchased from Fluka (Buchs, Switzerland). Folin-Ciocalteu's phenol reagent and potassium ferricyanide were obtained from Merck (Darmstadt, Germany). All other chemicals and solvents used were of analytical grade.

### 2.4. Phytochemical Analyses of Polyphenols

#### 2.4.1. TLC Analysis

Thin-layer chromatographic (TLC) analysis of phenolic acids and flavonoids was performed on precoated silica gel 60 F_254_ TLC plate (Merck, Germany) using diisopropyl ether–acetone–formic acid–water (50 : 30 : 10 : 10) as mobile phase. Polyphenolic compounds were detected under UV light at 365 nm after spraying plates with natural products-polyethylene glycol reagent (1% methanolic solution of 2-aminoethyl diphenylborinate and 5% ethanolic solution of PEG 4000) [16].

#### 2.4.2. Assay for Total Hydroxycinnamic Acids

Determination of total hydroxycinnamic acids was performed using method previously described by Štefan et al. [17]. The content of total hydroxycinnamic acids, expressed as rosmarinic acid, was calculated from the expression: (%) = *A* × 2.5/*m*, where *A* is the absorbance of the test solution at 505 nm and *m* is the mass of the sample, in grams.

#### 2.4.3. Assay for Total Flavonoids

The total flavonoid contents of six* Thymus* species were determined using aluminum chloride colorimetric method described in European Pharmacopoeia [18]. The percentage content of flavonoids, expressed as isoquercitrin, was calculated from the equation: (%) = *A* × 1.25/*m*, where *A* is the absorbance of the test solution at 425 nm and *m* is the mass of the sample, in grams.

#### 2.4.4. Assay for Total Tannins

Determination of total tannins was performed following the Folin-Ciocalteu method described in European Pharmacopoeia [18]. The percentage content of tannins, expressed as pyrogallol, was calculated from the following equation: (%) = 3.125 × (*A*_1_ − *A*_2_)/(*A*_3_ × *m*), where *A*_1_ is the absorbance of the solution containing total polyphenols, *A*_2_ is the absorbance of the solution containing polyphenols not adsorbed by hide powder, *A*_3_ is the absorbance of the test solution containing 0.05 mg of pyrogallol, and *m* is the mass of the sample, in grams.

#### 2.4.5. Assay for Procyanidins

Determination of procyanidin contents of selected* Thymus* species was done using European Pharmacopoeia method [18]. The percentage content of procyanidins was calculated as cyanidin chloride from the expression: (%) = *A* × 6.7/*m*, where *A* is the absorbance at 545 nm and *m* is the mass of the sample, in grams.

### 2.5. Evaluation of Antioxidant Activity

#### 2.5.1. DPPH Radical Scavenging Assay

The free radical scavenging activities of the samples were measured using the stable DPPH radical, according to the previously reported method [19]. The samples were assayed in the range of 0.2–100 *μ*g/mL. Trolox was used as a positive control. The capability to scavenge the DPPH radicals was calculated using the following equation: (%) = (1 − *A*_1_/*A*_0_) × 100, where *A*_0_ is the absorbance of the control reaction and *A*_1_ is the absorbance in the presence of the sample, corrected for the absorbance of sample itself.

#### 2.5.2. Nitric Oxide Radical Scavenging Assay

The nitric oxide (NO^•^) scavenging activity was determined according to the method described by Razali et al. [20] with a slight modification. Sodium nitroprusside in aqueous solution at physiological pH spontaneously generates nitric oxide which interacts with oxygen to produce nitrite ions that can be estimated using Griess reagent. Equal volumes of 10 mmol/L sodium nitroprusside in phosphate buffered-saline (pH 7.4) were mixed with different concentrations of the sample (6.25–400 *μ*g/mL) and incubated at room temperature for 120 min. After the incubation, 0.5 mL of the reaction mixture was mixed with 1.0 mL of 1% sulfanilamide in 5% phosphoric acid. After 5 min, 0.1% naphthylethylenediamine (1.0 mL) was added, the solution was mixed, and the absorbance was measured at 540 nm against the corresponding blank solution. Trolox was used as a standard. The NO^•^ scavenging activity was expressed as the percentage of inhibition according to the following equation: (%) = (1 − *A*_1_/*A*_0_) × 100, where *A*_0_ is the absorbance of the control without a sample and *A*_1_ is the absorbance in the presence of the sample.

#### 2.5.3. Reducing Power Assay

The reducing power of the studied* Thymus* plant extracts and their polyphenolic constituents were evaluated by the potassium ferricyanide reduction method previously described by Vladimir-Knežević et al. [21]. The effective concentrations of the samples in the reaction mixture were in the range of 0.2–100 *μ*g/mL. Trolox was used as a reference antioxidant compound. The sample concentration providing absorbance of 0.5 (IC_50_) was calculated from the graph of absorbance at 700 nm against sample concentration.

#### 2.5.4. Metal Ion Chelating Assay

The ability of samples to chelate iron(II) ions at different concentrations (37.5–600 *μ*g/mL) was estimated using the previously reported method [21]. The percentage of inhibition of ferrozine-Fe^2+^ complex formation was calculated using the formula given below: (%) = (1 − *A*_1_/*A*_0_) × 100, where *A*_0_ is the absorbance of the control and *A*_1_ is the absorbance in the presence of sample, corrected for the absorbance of sample itself.

#### 2.5.5. Lipid Peroxidation Assay

The ability of the extracts to inhibit lipid peroxidation was evaluated by the thiobarbituric acid reactive substances assay using Fe(II)–H_2_O_2_ system in bovine brain liposomes [19]. The extracts were tested in the range of 10–1000 *μ*g/mL, while rosmarinic acid and luteolin were tested at concentrations from 1.56 *μ*g/mL to 100 *μ*g/mL. Inhibition of lipid peroxidation in percent was calculated using the following equation: (%) = (1 − *A*_1_/*A*_0_) × 100, where *A*_0_ is the absorbance of the negative control reaction and *A*_1_ is the absorbance in the presence of the tested sample.

#### 2.5.6. Total Antioxidant Capacity Assay

The total antioxidant capacities of the tested extracts, rosmarinic acid, luteolin, and trolox (25–100 *μ*g/mL) were evaluated by the phosphomolybdenum method [22]. The antioxidant capacity of the sample was expressed as equivalents of ascorbic acid (AAE), utilizing a calibration curve of ascorbic acid in the concentration range 0.8–100 *μ*g/mL.

### 2.6. Acetylcholinesterase Inhibition Assay

The AChE inhibitory activities of* Thymus* extracts were determined using modified Ellman's colorimetric method described by Conforti et al. [23]. In brief, 1900 *μ*L of 50 mM Tris-HCl buffer (pH 8.0), 40 *μ*L of 0.02 U/mL AChE, and 20 *μ*L of tested solution (0.25–1 mg/mL) were mixed and preincubated for 30 min at 4°C. The reaction was then initiated with the addition of 20 *μ*L of 10 mM DTNB and 20 *μ*L of 12 mM ATChI. AChE activity was determined spectrophotometrically through measuring the change in absorbance of an assay solution at 412 nm over a period of 10 min. Galantamine (0.03–1 *μ*g/mL) was used as a positive control. Percentage enzyme inhibition was calculated by comparing the enzymatic activity with and without inhibitor using the formula (*E* − *S*)/*E* × 100, where *E* is the activity of enzyme without a test sample and *S* is the activity of enzyme in the presence of the sample.

### 2.7. Statistical Analysis

All experiments were carried out in triplicate, and the results are expressed as mean ± standard deviation (SD). The concentrations of the samples that provide 50% inhibition (IC_50_) were obtained by interpolation from linear regression analysis. The results were tested for normal distribution by Kolmogorov-Smirnov test. Correlations were assessed using Pearson's correlation coefficient (*r*). One way analysis of variance (ANOVA) coupled with the Holm-Sidak post hoc tests was used to compare the data and to identify means with significant differences. Differences with *P* < 0.05 were considered statistically significant. The data analysis was performed using Microsoft Excel 2007 software and SigmaPlot for Windows (version 12.0).

## 3. Results and Discussion

### 3.1. Phytochemical Analysis of Polyphenolic Compounds

Plant polyphenols have gained increasing scientific interest due to their potent antioxidant properties and their remarkable effects in the prevention of various oxidative stress-associated diseases [24]. Hence, qualitative and quantitative analyses of polyphenolic compounds in plants are important in understanding their medicinal value.

The presence of phenolic acids and flavonoids in* Thymus* extracts was detected by thin-layer chromatography (TLC) in comparison with related standards. Rosmarinic acid with *R*_*f*_ value of 0.81 was identified in all tested extracts while luteolin-7-O-glucoside (*R*_*f*_ = 0.43) was detected in the extracts of* T. longicaulis* and* T. vulgaris*. The chromatogram also showed several other yellowish fluorescent zones having *R*_*f*_ values between 0.13 and 0.71. According to the previously reported data, these compounds are most likely luteolin derivatives [10, 25, 26].

The contents of different polyphenolic subclasses in aerial plant parts were determined spectrophotometrically and the results are presented in Table 1. The most abundant compounds were hydroxycinnamic acids (3.35–6.17%), followed by tannins (0.77–1.59%) and flavonoids (0.15–0.42%). The percentage of procyanidin group of tannins varied between 0.21% (*T. vulgaris*) and 0.70% (*T. longicaulis*). Aerial parts of* T. pulegioides* and* T. longicaulis* contained the highest amount of hydroxycinnamic acids, 6.17% and 5.41%, respectively. These two* Thymus* species also had the highest contents of tannins, 1.59% and 1.33%, respectively. The richest species regarding the flavonoid content were found to be* T. pulegioides* (0.42%),* T. longicaulis* (0.40%), and* T. serpyllum* subsp.* serpyllum* (0.40%).

### 3.2. Antioxidant Activities of* Thymus* Ethanolic Extracts

The antioxidant activity may be due to different mechanisms, such as prevention of chain initiation, decomposition of peroxides, prevention of continued hydrogen abstraction, free radical scavenging, reducing capacity, and binding of prooxidant metal ions [27]. Therefore, in the present study, six different assays were employed in order to evaluate the antioxidant properties of the ethanolic extracts of selected* Thymus* species as well as elucidate its mode of action.

DPPH assay has been widely used to evaluate the free radical scavenging ability of the plant extracts and polyphenolic compounds. The tested* Thymus* extracts and pure compounds at different concentrations (0.4–25 *μ*g/mL) significantly inhibited DPPH^•^ in a concentration dependent manner (Table 2). The activities of plant extracts were 11–28%, 23–52%, and 52–85% at 1.56 *μ*g/mL, 3.13 *μ*g/mL, and 6.25 *μ*g/mL, respectively. At the mentioned concentrations,* T. serpyllum* subsp.* serpyllum* as well as commercial sample of* T. vulgaris* were the least effective. Rosmarinic acid and luteolin at concentrations up to 3.13 *μ*g/mL showed the highest radical scavenging effectiveness (56% and 50% at 0.8 *μ*g/mL, resp.). Interestingly, at concentrations ≥ 12.5 *μ*g/mL, activities of most* Thymus* species were comparable to that of luteolin. DPPH radical scavenging activities of the tested samples were assessed using IC_50_ values (Table 7) which are inversely related to their antioxidant abilities. The obtained IC_50_ values of studied* Thymus* extracts were in the range 3.01–6.01 *μ*g/mL. The scavenging effects of the extracts decreased in order of* T. longicaulis* >* T. praecox* subsp.* polytrichus* >* T. pulegioides*,* T. striatus* >* T. vulgaris* >* T. serpyllum* subsp.* serpyllum*. Although there have been several reports on DPPH radical scavenging activity of polar extracts of these* Thymus* species [26–33], the lack of standardization of the results makes it difficult to compare their antioxidant strength [34]. The activities of rosmarinic acid and luteolin did not differ significantly (IC_50_ = 0.66 *μ*g/mL and IC_50_ = 0.73 *μ*g/mL, resp., *P* = 0.612) and were found to be higher than that of trolox (IC_50_ = 1.67 *μ*g/mL), indicating their high antioxidant potency in terms of electron or hydrogen atom-donating capacity.

NO is an important signaling molecule involved in the regulation of various physiological processes including neurotransmission, vascular homeostasis, antimicrobial defense, and antitumor activities. However, it has been demonstrated that increased NO production may also contribute to the pathogenesis of neurodegenerative human diseases and inflammatory tissue injury. The contribution of NO to the oxidative damage is increasingly becoming evident due to the fact that NO can react with superoxide to form highly reactive nitrogen species [35]. As can be seen in Table 3, all tested* Thymus* extracts inhibited nitrite formation in a concentration dependent manner. They scavenged NO^•^ by 16–45%, 37–58%, and 54–72% at 50 *μ*g/mL, 100 *μ*g/mL, and 200 *μ*g/mL, respectively. At these concentrations,* T. longicaulis* and* T. pulegioides* showed the highest activity, while* T. serpyllum* subsp.* serpyllum* demonstrated the weakest effect. Rosmarinic acid and luteolin inhibited the formation of NO^•^ by 58% already at 25 *μ*g/mL. Comparing obtained IC_50_ values (Table 7), the effectiveness of plant extracts as NO^•^ scavengers was in the following descending order:* T. longicaulis*,* T. pulegioides* >* T. striatus*, and* T. vulgaris* >* T. praecox* subsp.* polytrichus* >* T. serpyllum* subsp.* serpyllum*. The IC_50_ values of the most potent* T. longicaulis* and* T. pulegioides* were 71.57 *μ*g/mL and 69.77 *μ*g/mL, respectively. Rosmarinic acid (IC_50_ = 15.67 *μ*g/mL) and luteolin (IC_50_ = 18.31 *μ*g/mL) demonstrated the greatest NO^•^ scavenging activity, even significantly higher (*P* < 0.001) than trolox (IC_50_ = 53.91 *μ*g/mL), and these results were in accordance with the findings obtained by DPPH assay.

The reducing capacity of a compound is an important mechanism of antioxidant action and may serve as a significant indicator of its potential antioxidant activity. Many studies have indicated that the antioxidant effect is related to the presence of reductones which were reported to be terminators of free radical chain reactions by donating hydrogen atom. Furthermore, reductones can also react with certain precursors of peroxide, thus preventing peroxide formation [36].  Table 4 shows the reducing power for different concentrations (0.8–50 *μ*g/mL) of* Thymus* extracts, rosmarinic acid, luteolin, and trolox as a positive control. Our results demonstrated that all plant extracts possessed strong ability to reduce iron(III) ions (IC_50_ = 11.39–15.10 *μ*g/mL) and the reducing power increased with a concentration in a strongly linear manner (*R* ^2^ = 0.9840–0.9962). In this assay,* T. pulegioides* (IC_50_ = 11.39 *μ*g/mL) and* T. longicaulis* (IC_50_ = 11.84 *μ*g/mL) showed stronger effect than other tested extracts (IC_50_ = 14.41–15.10 *μ*g/mL) which did not differ significantly from one another (Table 7). These results were not consistent with previous research on reducing activities of the ethanolic and methanolic extracts of* T. longicaulis* subsp.* longicaulis* var.* longicaulis* and two varieties of* T. praecox* growing in Turkey [27, 32, 33] which showed much weaker ability to reduce iron(III) ions. Rosmarinic acid and luteolin with IC_50_ values of 2.67 *μ*g/mL and 4.51 *μ*g/mL, respectively, demonstrated the strongest reducing properties, even significantly stronger (*P* < 0.001) than trolox (IC_50_ = 6.64 *μ*g/mL).

An imbalance of metal homeostasis in the brain is thought to play an important role in the pathogenesis of AD. Amyloid *β*-peptide, as a pathological hallmark of AD, is considered a strong redox active agent that can efficiently generate reactive oxygen species in the presence of the transition metals such as copper and iron [5]. Additionally, iron is known as the most important lipid oxidation prooxidant due to its high reactivity. The ferrous state of iron accelerates lipid oxidation by breaking down hydrogen peroxide and lipid peroxides to reactive free radicals via the Fenton reaction [32]. Therefore, metal-chelating properties of the polyphenols indicate their antioxidant activity. The ability of tested* Thymus* extracts to compete with ferrozine for iron(II) ions was studied, and the obtained results are presented in Table 5, while the corresponding IC_50_ values are given in Table 7. All* Thymus* extracts displayed ferrous ion chelating properties at concentrations higher than 150 *μ*g/mL, apart from* T. vulgaris* extract which exhibited its effect already at 37.5 *μ*g/mL. At all tested concentrations,* T. vulgaris* demonstrated the strongest chelating effect (7–96%). The activities of other plant extracts at 150 *μ*g/mL, 300 *μ*g/mL, and 600 *μ*g/mL were 1–15%, 39–58%, and 74–79%, respectively. Among these five extracts, only* T. longicaulis* and* T. pulegioides* showed over 50% chelating activity at concentration of 300 *μ*g/mL. The effectiveness of plant extracts decreased in the following order:* T. vulgaris* >* T. longicaulis*,* T. pulegioides* >* T. serpyllum* subsp.* serpyllum*,* T. striatus* >* T. praecox* subsp.* polytrichus*, with IC_50_ values ranging between 125.91 *μ*g/mL and 388.77 *μ*g/mL. The chelating abilities of tested* Thymus* extracts were much lower compared to reference EDTA (IC_50_ = 5.34 *μ*g/mL). Presented results also confirmed previous findings on polar extracts of* T. longicaulis* subsp.* longicaulis* var.* longicaulis* and two varieties of* T. praecox* reported to have ferrous ion chelating activity [27, 32, 33]. Rosmarinic acid and luteolin showed no activity at tested concentrations. These findings indicate that chelating activities of* Thymus* ethanolic extracts could be attributed to the other phenolic constituents.

The high lipid content of cell membranes makes them one of the main targets of the reactive oxygen species. Lipid peroxidation, as a well-established mechanism of cellular injury, can be used as an indicator of oxidative stress in the cells and tissues. It has been implicated in the pathogenesis of various diseases such as cardiovascular diseases, cancer, and neurodegenerative disorders as well as aging processes [37]. All investigated* Thymus* extracts inhibited lipid peroxidation in a concentration dependent manner (Table 6). The activities of plant extracts at concentrations of 10 *μ*g/mL and 100 *μ*g/mL were in the ranges 32–40% and 56–76%, respectively. Among investigated species,* T. longicaulis* (IC_50_ = 34.30 *μ*g/mL) and* T. pulegioides* (IC_50_ = 34.83 *μ*g/mL) exhibited once again the most powerful antioxidant effect, comparable to that of rosmarinic acid (IC_50_ = 21.07 *μ*g/mL) (Table 7). The IC_50_ values obtained for other four extracts were in the range 63.01–80.00 *μ*g/mL, without significant difference between them. In contrast to previously presented results which revealed very similar antioxidant activity of rosmarinic acid and luteolin, in this assay, luteolin was found to be the most potent antioxidant against lipid peroxidation with IC_50_ value of 2.03 *μ*g/mL.

**Figure 1:**
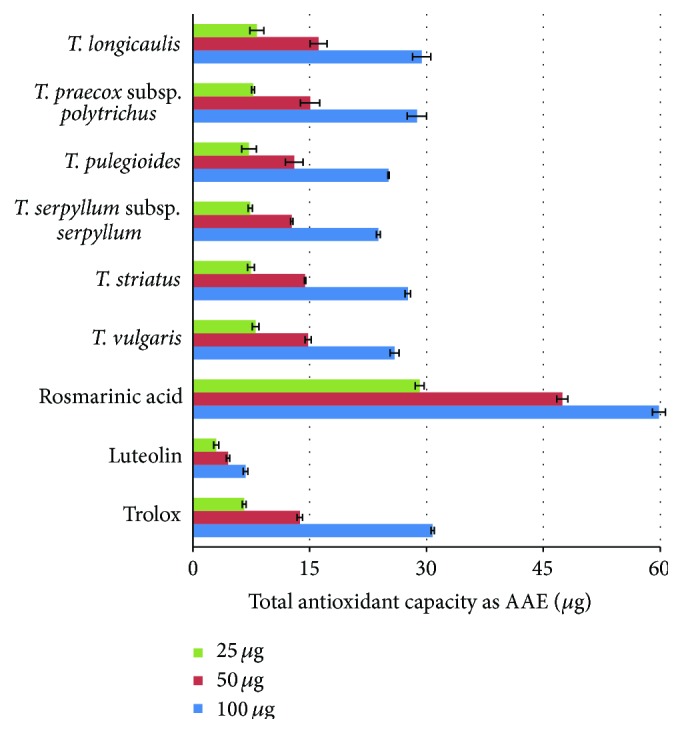
Total antioxidant capacities of* Thymus* ethanolic extracts, polyphenolic compounds, and reference antioxidant.

Total antioxidant capacities of* Thymus* ethanolic extracts, rosmarinic acid, luteolin, and trolox were expressed as ascorbic acid equivalents (AAE) and are presented in Figure 1 and Table 7. All investigated plant samples were active in a concentration dependent manner with total antioxidant capacities ranging between 238.16 mg AAE/g and 293.82 mg AAE/g. Their effectiveness decreased in the following order:* T. longicaulis*,* T. praecox* subsp.* polytrichus* ≥* T. striatus* >* T. pulegioides*,* T. vulgaris* ≥* T. serpyllum* subsp.* serpyllum*. The activity of* T. longicaulis* was comparable to that of trolox. Rosmarinic acid was found to have much higher total antioxidant capacity (598.34 mg AAE/g) than trolox (307.89 mg AAE/g). Luteolin showed the lowest activity in comparison to all tested samples (67.72 mg AAE/g).

For the first time, this paper provides comprehensive comparative data on the antioxidant activities of selected* Thymus* species growing wild in Croatia. According to our findings, rosmarinic acid was confirmed as important contributor to the overall antioxidant effectiveness of* Thymus* species, as previously suggested by other researchers. Our results were also consistent with earlier studies which pointed out plants belonging to* Thymus* genus from different origin as a rich source of antioxidant polyphenols. In these studies, correlations were found between antioxidant effects of* Thymus* extracts and their flavonoid and phenolic acid contents [25, 38–40].

### 3.3. Antiacetylcholinesterase Activities of* Thymus* Ethanolic Extracts

**Figure 2:**
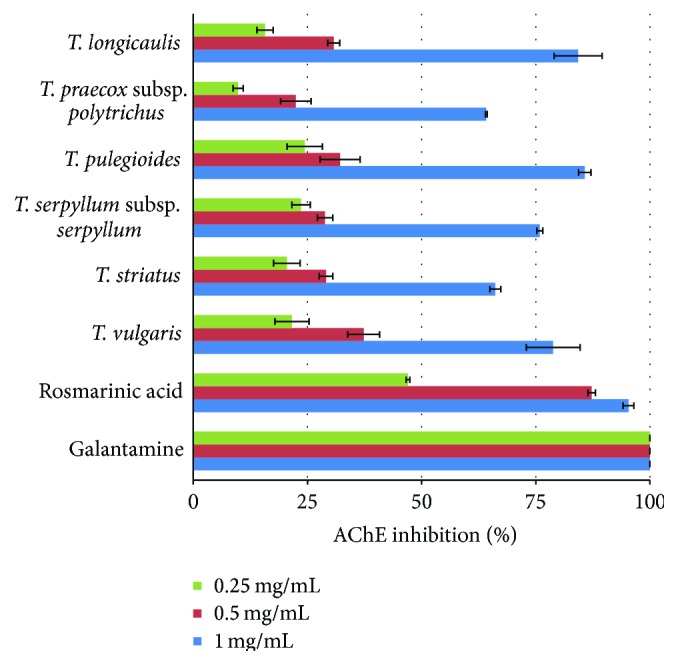
Acetylcholinesterase (AChE) inhibitory activities of* Thymus* ethanolic extracts, rosmarinic acid, and galantamine as reference drug.

A variety of plants and their polyphenolic constituents have been reported to show AChE inhibitory activity and thus could be useful in the prevention and treatment of neurodegenerative disorders such as AD [7]. In this study,* Thymus* ethanolic extracts were tested for their inhibitory activity against AChE at concentrations of 0.25, 0.5 and 1 mg/mL using Ellman's colorimetric assay. All tested extracts exhibited AChE inhibitory activity in a dose dependent manner (Figure 2). The values of 10−28%, 23−39%, and 64−86% were obtained for tested concentrations of 0.25, 0.5, and 1 mg/mL, respectively. Among tested plant extracts,* T. longicaulis*,* T. pulegioides*, and* T. vulgaris* showed the most potent inhibitory activity against AChE with IC_50_ values 656.06–667.91 *μ*g/mL, with no significant difference between them. The AChE inhibitory activities of three remaining plant extracts were in the following descending order:* T. serpyllum* subsp.* serpyllum* >* T. striatus* >* T. praecox* subsp.* polytrichus*. Obtained results for* T. serpyllum* subsp.* serpyllum* (IC_50_ = 742.11 *μ*g/mL) confirmed previous findings on anti-AChE activity of* T. serpyllum* ethanolic extract [30]. Studies on AChE inhibitory properties of the ethanolic extracts of* T. praecox* [41] and* T. praecox* subsp.* caucasicus* var.* caucasicus* [33] showed no activity, which is not in accordance with our evidence on anti-AChE activity of* T. praecox* subsp.* polytrichus* (IC_50_ = 837.96 *μ*g/mL). Rosmarinic acid inhibited 47% and 87% of AChE activity at 0.25 and 0.5 mg/mL, respectively. AChE inhibition by luteolin was not determined because of its insolubility at tested concentrations. Galantamine, as a reference AChE inhibitor used, demonstrated the strongest effect comparing with the tested samples (IC_50_ = 0.122 ± 0.004 *μ*g/mL).

As suggested in our previous work [22], considerable antioxidant abilities and AChE inhibition of most Lamiaceae species could be attributed to the high level of polyphenols, particularly rosmarinic acid. Hence, the influence of polyphenol contents on antioxidant and AChE inhibitory properties of the tested* Thymus* species was evaluated. A very strong correlation was observed only between the content of polyphenols and reducing power (*r* = −0.920, *P* = 0.009), while no significant correlation was found between polyphenol levels and all other effects of tested extracts proved in this study. These findings are probably due to the presence of terpene compounds which are known to possess antioxidant and anti-AChE properties [42].

## 4. Conclusions

Considering a very large body of evidence supporting the involvement of oxidative stress in neurodegenerative pathologies as well as the fact that AChE inhibition is up to now the most effective therapeutic approach to dementia, this study aimed to evaluate the neuroprotective potential of six* Thymus* species through investigation of their antioxidant and anti-AChE activities. Our results showed that all tested plant species possess strong antioxidant properties acting as radical scavengers, reducing agents, chelating transition metals, and inhibiting lipid peroxidation. Also, all tested extracts exhibited anti-AChE activity in a dose dependent manner. The obtained results provide evidence that the investigated* Thymus* species containing both terpenes and polyphenols could be used as promising therapeutic agents for neurodegenerative disorders. These findings have inspired our current study for isolation and structure elucidation of active components of* Thymus* extracts.

## Figures and Tables

**Table 1:** Yield percentages of the ethanolic extracts and contents of total hydroxycinnamic acids, flavonoids, tannins, and procyanidins in aerial parts of selected *Thymus* species.

Plant species	Extract yield^*∗*^	Content (%)			
		Hydroxycinnamic acids	Flavonoids	Tannins	Procyanidins
*T. longicaulis *	21.34	5.41 ± 0.09^a^	0.40 ± 0.004^a^	1.33 ± 0.02^a^	0.70 ± 0.015^a^
*T. praecox *subsp.* polytrichus *	20.06	4.39 ± 0.01^b^	0.24 ± 0.010^b^	0.93 ± 0.05^b^	0.29 ± 0.002^b^
*T. pulegioides *	22.54	6.17 ± 0.07^c^	0.42 ± 0.003^c^	1.59 ± 0.04^c^	0.41 ± 0.002^c^
*T. serpyllum* subsp. *serpyllum *	19.22	4.36 ± 0.11^b^	0.40 ± 0.006^a^	1.06 ± 0.08^d^	0.45 ± 0.004^d^
*T. striatus *	15.54	3.35 ± 0.10^d^	0.15 ± 0.003^d^	0.77 ± 0.07^e^	0.26 ± 0.003^e^
*T. vulgaris *	18.28	3.58 ± 0.03^e^	0.24 ± 0.006^b^	0.98 ± 0.03^b,d^	0.21 ± 0.002^f^

^*∗*^Extract yield expressed as percentage weight of air-dried plant material.

Mean values in the same column followed by different letters are significantly different from each other (*P* < 0.05).

**Table 2:** DPPH radical scavenging effects of *Thymus* ethanolic extracts, polyphenolic compounds, and reference antioxidant.

Samples	DPPH^∙^ scavenging activity (%) ± SD						
	0.39 *μ*g/mL	0.78 *μ*g/mL	1.56 *μ*g/mL	3.13 *μ*g/mL	6.25 *μ*g/mL	12.5 *μ*g/mL	25 *μ*g/mL
*T. longicaulis *	12.82 ± 3.02^a^	17.74 ± 1.51^a^	27.78 ± 0.60^a^	51.71 ± 0.03^a^	84.62 ± 3.02^a^	87.18 ± 0.09^a^	87.82 ± 1.51^a^
*T. praecox *subsp.* polytrichus *	10.84 ± 2.73^a,b^	13.52 ± 2.12^b^	27.68 ± 2.12^a^	47.00 ± 2.73^b^	83.69 ± 0.61^a^	86.70 ± 0.58^a^	86.70 ± 0.61^a,b^
*T. pulegioides *	8.61 ± 1.87^a,c^	12.25 ± 0.05^b^	20.20 ± 0.94^b^	38.58 ± 0.23^c^	73.18 ± 0.94^b^	83.94 ± 0.23^a,b^	87.25 ± 1.64^a^
*T. serpyllum* subsp. *serpyllum *	2.05 ± 0.48^d^	8.39 ± 2.18^c^	11.13 ± 0.24^c^	22.95 ± 4.36^d^	52.23 ± 4.12^c^	82.02 ± 1.70^b,c^	84.42 ± 0.73^b^
*T. striatus *	7.05 ± 1.25^b,c,d^	9.91 ± 0.93^b,c^	19.82 ± 1.87^b^	40.53 ± 0.62^c^	75.11 ± 0.31^b^	86.34 ± 2.49^a^	87.67 ± 0.03^a^
*T. vulgaris *	4.75 ± 0.65^c,d^	6.58 ± 1.42^c^	14.90 ± 1.01^d^	29.56 ± 0.26^e^	55.27 ± 0.69^c^	79.73 ± 0.27^c^	80.95 ± 0.13^c^
Rosmarinic acid	37.45 ± 2.07^e^	56.49 ± 1.78^d^	89.12 ± 0.02^e^	93.10 ± 0.30^f^	94.98 ± 0.02^d^	94.98 ± 0.09^d^	95.19 ± 0.30^d^
Luteolin	25.00 ± 1.61^f^	50.19 ± 1.87^e^	82.01 ± 0.80^f^	84.47 ± 1.07^g^	84.85 ± 0.14^a^	85.80 ± 1.87^a^	87.12 ± 1.61^a^
Trolox	18.98 ± 2.82^g^	24.44 ± 0.13^f^	47.19 ± 2.64^g^	87.22 ± 0.60^g^	93.98 ± 0.03^d^	95.68 ± 0.29^d^	96.62 ± 0.02^d^

Mean values in the same column followed by different letters are significantly different from each other (*P* < 0.05).

**Table 3:** NO radical scavenging effects of *Thymus *ethanolic extracts, polyphenolic compounds, and reference antioxidant.

Samples		NO^∙^ scavenging activity (%) ± SD					
	6.25 *μ*g/mL	12.5 *μ*g/mL	25 *μ*g/mL	50 *μ*g/mL	100 *μ*g/mL	200 *μ*g/mL	400 *μ*g/mL
*T. longicaulis *	7.29 ± 1.05^a^	15.47 ± 1.28^a^	29.62 ± 2.75^a^	44.17 ± 1.47^a^	57.64 ± 1.10^a^	72.25 ± 0.11^a^	75.01 ± 0.95^a^
*T. praecox *subsp.* polytrichus *	na	0.23 ± 0.03^b^	10.16 ± 0.46^b^	26.03 ± 1.52^b^	43.02 ± 1.72^b^	60.77 ± 0.13^b^	68.22 ± 0.03^b^
*T. pulegioides *	0.37 ± 0.05^b^	11.64 ± 0.90^c^	29.40 ± 1.10^a^	44.58 ± 2.19^a,g^	58.01 ± 0.37^a^	70.40 ± 0.46^a^	70.89 ± 1.54^c^
*T. serpyllum* subsp. *serpyllum *	na	na	2.73 ± 1.46^c^	16.38 ± 1.20^c^	37.08 ± 1.66^c^	53.92 ± 1.26^c^	62.38 ± 0.80^d^
*T. striatus *	7.73 ± 0.78^a,c^	21.19 ± 0.98^d^	28.73 ± 2.16^a^	39.76 ± 2.04^d^	52.17 ± 1.17^d^	63.24 ± 2.19^b^	66.12 ± 1.26^e^
*T. vulgaris *	2.16 ± 0.82^b^	14.96 ± 1.23^a,c^	24.42 ± 0.35^d^	39.02 ± 0.72^d^	50.52 ± 0.74^d^	60.96 ± 1.17^b^	63.73 ± 0.55^d^
Rosmarinic acid	10.86 ± 3.09^c^	47.20 ± 0.23^e^	58.40 ± 2.23^e^	68.19 ± 0.27^e^	73.75 ± 0.33^e^	76.23 ± 0.76^d^	77.22 ± 0.21^f^
Luteolin	1.57 ± 0.21^b^	43.10 ± 3.95^f^	57.64 ± 2.75^e^	64.34 ± 0.62^f^	70.95 ± 1.92^f^	76.25 ± 1.06^d^	80.31 ± 0.30^g^
Trolox	na	5.87 ± 1.20^g^	20.06 ± 0.02^f^	47.39 ± 0.14^g^	80.70 ± 0.54^g^	83.62 ± 0.62^e^	84.86 ± 1.23^h^

na: no activity. Mean values in the same column followed by different letters are significantly different from each other (*P* < 0.05).

**Table 4:** Ferric reducing power of *Thymus *ethanolic extracts, polyphenolic compounds, and reference antioxidant.

Samples	Absorbance at 700 nm ± SD						
	0.78 *μ*g/mL	1.56 *μ*g/mL	3.13 *μ*g/mL	6.25 *μ*g/mL	12.5 *μ*g/mL	25 *μ*g/mL	50 *μ*g/mL
*T. longicaulis *	0.065 ± 0.001^a,b^	0.105 ± 0.001^a,b^	0.172 ± 0.003^a,b^	0.301 ± 0.001^a^	0.534 ± 0.008^a^	0.946 ± 0.011^a^	1.619 ± 0.008^a^
*T. praecox *subsp.* polytrichus *	0.060 ± 0.004^a,c^	0.090 ± 0.004^a,c^	0.147 ± 0.015^a^	0.242 ± 0.019^b^	0.432 ± 0.018^b^	0.782 ± 0.019^b^	1.340 ± 0.014^b^
*T. pulegioides *	0.070 ± 0.001^b^	0.109 ± 0.001^b^	0.179 ± 0.002^b^	0.319 ± 0.005^c^	0.547 ± 0.004^a^	0.961 ± 0.023^a^	1.541 ± 0.029^c^
*T. serpyllum* subsp. *serpyllum *	0.060 ± 0.006^a,c^	0.090 ± 0.007^a,c^	0.147 ± 0.015^a^	0.254 ± 0.019^b^	0.442 ± 0.023^b^	0.809 ± 0.008^b^	1.359 ± 0.064^b^
*T. striatus *	0.055 ± 0.001^c^	0.085 ± 0.004^c^	0.143 ± 0.006^a^	0.249 ± 0.006^d^	0.447 ± 0.010^b^	0.795 ± 0.017^b^	1.439 ± 0.017^d^
*T. vulgaris *	0.062 ± 0.001^a,b,c^	0.094 ± 0.003^a,b,c^	0.149 ± 0.005^a,b^	0.254 ± 0.001^b^	0.464 ± 0.014^b^	0.797 ± 0.001^b^	1.376 ± 0.005^b,d^
Rosmarinic acid	0.173 ± 0.005^d^	0.321 ± 0.013^d^	0.581 ± 0.015^c^	1.055 ± 0.028^e^	1.777 ± 0.065^c^	3.251 ± 0.086^c^	3.584 ± 0.010^e^
Luteolin	0.142 ± 0.006^e^	0.228 ± 0.008^e^	0.390 ± 0.012^d^	0.641 ± 0.037^f^	1.132 ± 0.026^d^	1.782 ± 0.045^d^	3.568 ± 0.038^e^
Trolox	0.089 ± 0.002^f^	0.148 ± 0.007^f^	0.240 ± 0.016^e^	0.432 ± 0.018^g^	0.903 ± 0.025^e^	1.496 ± 0.021^e^	2.941 ± 0.001^f^

Mean values in the same column followed by different letters are significantly different from each other (*P* < 0.05).

**Table 5:** Ferrous ion chelating capacities of *Thymus *ethanolic extracts.

Samples	Iron(II) chelating ability ± SD				
	37.5 *μ*g/mL	75 *μ*g/mL	150 *μ*g/mL	300 *μ*g/mL	600 *μ*g/mL
*T. longicaulis *	na	1.92 ± 0.03^a^	15.13 ± 2.71^a^	58.05 ± 2.17^a^	77.30 ± 0.68^a,c^
*T. praecox *subsp.* polytrichus *	na	na	1.30 ± 0.95^b^	39.19 ± 0.48^b^	75.72 ± 0.25^a,b^
*T. pulegioides *	na	na	9.32 ± 3.06^a,c^	57.34 ± 0.93^a^	77.40 ± 1.07^a,c^
*T. serpyllum* subsp. *serpyllum *	na	na	2.47 ± 0.44^b^	46.04 ± 0.44^c^	74.41 ± 1.89^b^
*T. striatus *	na	na	3.07 ± 4.34^b,c^	46.44 ± 0.42^c^	78.61 ± 0.84^c^
*T. vulgaris *	6.85 ± 3.07	19.61 ± 1.52^b^	64.38 ± 1.47^d^	92.71 ± 1.38^e^	95.70 ± 0.03^d^

na: no activity. Mean values in the same column followed by different letters are significantly different from each other (*P* < 0.05).

**Table 6:** Inhibition of lipid peroxidation by *Thymus *ethanolic extracts.

Samples	Inhibition of lipid peroxidation (%) ± SD			
	10 *μ*g/mL	100 *μ*g/mL	500 *μ*g/mL	1000 *μ*g/mL
*T. longicaulis *	40.20 ± 3.38^a^	76.26 ± 1.80^a^	89.75 ± 1.34^a^	97.84 ± 1.24^a^
*T. praecox *subsp.* polytrichus *	32.78 ± 1.89^b^	55.73 ± 5.12^b^	82.56 ± 6.46^a^	88.03 ± 3.82^b^
*T. pulegioides *	40.03 ± 3.37^a^	75.94 ± 1.79^a^	89.38 ± 1.33^a^	97.43 ± 1.23^a^
*T. serpyllum* subsp. *serpyllum *	32.91 ± 1.90^b^	55.45 ± 5.87^b^	82.40 ± 7.19^a^	88.40 ± 3.84^b^
*T. striatus *	31.89 ± 0.08^b^	63.68 ± 8.11^a,b^	91.29 ± 4.56^a^	98.56 ± 1.59^a^
*T. vulgaris *	31.76 ± 0.08^b^	59.42 ± 2.42^b^	90.92 ± 4.54^a^	98.15 ± 1.58^a^

Mean values in the same column followed by different letters are significantly different from each other (*P* < 0.05).

**Table 7:** Comparative overview of IC_50 _values as well as total antioxidant capacities of *Thymus *ethanolic extracts, polyphenolic compounds, and reference antioxidants.

Samples	IC_50 _(*μ*g/mL)					Total antioxidant capacity (mg AAE/g)^*∗*^
	DPPH radical scavenging activity	NO radical scavenging activity	Reducing power	Iron chelating activity	Inhibition of lipid peroxidation	
*T. longicaulis*	3.01 ± 0.02^a^	71.57 ± 4.85^a^	11.84 ± 0.11^a^	271.85 ± 7.94^a^	34.30 ± 7.37^a^	293.82 ± 11.64^a,b^
*T. praecox *subsp.* polytrichus*	3.41 ± 0.16^b^	139.04 ± 5.69^b^	15.10 ± 0.64^b^	388.77 ± 3.28^b^	78.74 ± 7.08^b^	287.72 ± 12.52^b,c^
*T. pulegioides*	4.18 ± 0.02^c^	69.77 ± 4.38^a^	11.39 ± 0.07^a^	277.10 ± 1.03^a^	34.83 ± 7.36^a^	251.26 ± 0.75^d,e^
*T. serpyllum* subsp. *serpyllum*	6.01 ± 0.44^d^	176.59 ± 8.09^c^	14.55 ± 0.57^b^	342.04 ± 6.89^c^	80.00 ± 9.93^b^	238.16 ± 2.50^d^
*T. striatus*	4.06 ± 0.04^c^	91.09 ± 5.34^d^	14.68 ± 0.38^b^	333.14 ± 4.39^c^	63.01 ± 7.44^b^	276.04 ± 3.38^c^
*T. vulgaris*	5.60 ± 0.07^e^	97.93 ± 2.93^d^	14.41 ± 0.19^b^	125.91 ± 2.50^d^	69.60 ± 5.14^b^	257.87 ± 5.76^e^
Rosmarinic acid	0.66 ± 0.03^f^	15.67 ± 0.44^e^	2.67 ± 0.10^c^	na	21.07 ± 1.63^a^	598.34 ± 8.26^f^
Luteolin	0.73 ± 0.11^f^	18.31 ± 1.02^e^	4.51 ± 0.07^d^	na	2.03 ± 0.23^c^	67.72 ± 3.00^g^
Trolox	1.67 ± 0.09^g^	53.91 ± 0.13^f^	6.64 ± 0.36^e^	na	—	307.89 ± 2.00^a^
EDTA	—	—	—	5.34 ± 0.10^e^	—	—

^*∗*^Results are calculated for sample concentration of 100 *μ*g/mL.

—: not tested; na: no activity. Mean values in the same column followed by different letters are significantly different from each other (*P* < 0.05).
